# Binding Specificity of ASHH2 CW Domain Toward H3K4me1 Ligand Is Coupled to Its Structural Stability Through Its α1-Helix

**DOI:** 10.3389/fmolb.2022.763750

**Published:** 2022-04-13

**Authors:** Maxim S. Bril’kov, Olena Dobrovolska, Øyvind Ødegård-Fougner, Diana C. Turcu, Øyvind Strømland, Jarl Underhaug, Rein Aasland, Øyvind Halskau

**Affiliations:** ^1^ Department of Biological Sciences, University of Bergen, Bergen, Norway; ^2^ Department of Pharmacy, University of Tromsø, Tromsø, Norway; ^3^ Department of Molecular Cell Biology, Institute for Cancer Research, The Norwegian Radium Hospital, Oslo, Norway; ^4^ Department of Biomedicine, University of Bergen, Bergen, Norway; ^5^ Department of Chemistry, University of Bergen, Bergen, Norway; ^6^ Department of Biosciences, University of Oslo, Oslo, Norway

**Keywords:** zinc-finger, histone methylation, histone modification reader, chromatin modification, NMR structure, SDG8, EFS, SET8

## Abstract

The CW domain binds to histone tail modifications found in different protein families involved in epigenetic regulation and chromatin remodeling. CW domains recognize the methylation state of the fourth lysine on histone 3 and could, therefore, be viewed as a reader of epigenetic information. The specificity toward different methylation states such as me1, me2, or me3 depends on the particular CW subtype. For example, the CW domain of ASHH2 methyltransferase binds preferentially to H3K4me1, and MORC3 binds to both H3K4me2 and me3 modifications, while ZCWPW1 is more specific to H3K4me3. The structural basis for these preferential bindings is not well understood, and recent research suggests that a more complete picture will emerge if dynamical and energetic assessments are included in the analysis of interactions. This study uses fold assessment by NMR in combination with mutagenesis, ITC affinity measurements, and thermal denaturation studies to investigate possible couplings between ASHH2 CW selectivity toward H3K4me1 and the stabilization of the domain and loops implicated in binding. The key elements of the binding site—the two tryptophans and the α1-helix form and maintain the binding pocket— were perturbed by mutagenesis and investigated. Results show that the α1-helix maintains the overall stability of the fold via the I915 and L919 residues and that the correct binding consolidates the loops designated as η1 and η3, as well as the C-terminal. This consolidation is incomplete for H3K4me3 binding to CW, which experiences a decrease in overall thermal stability on binding. Loop mutations not directly involved in the binding site, nonetheless, affect the equilibrium positions of the key residues.

## Introduction

**FIGURE 1:**
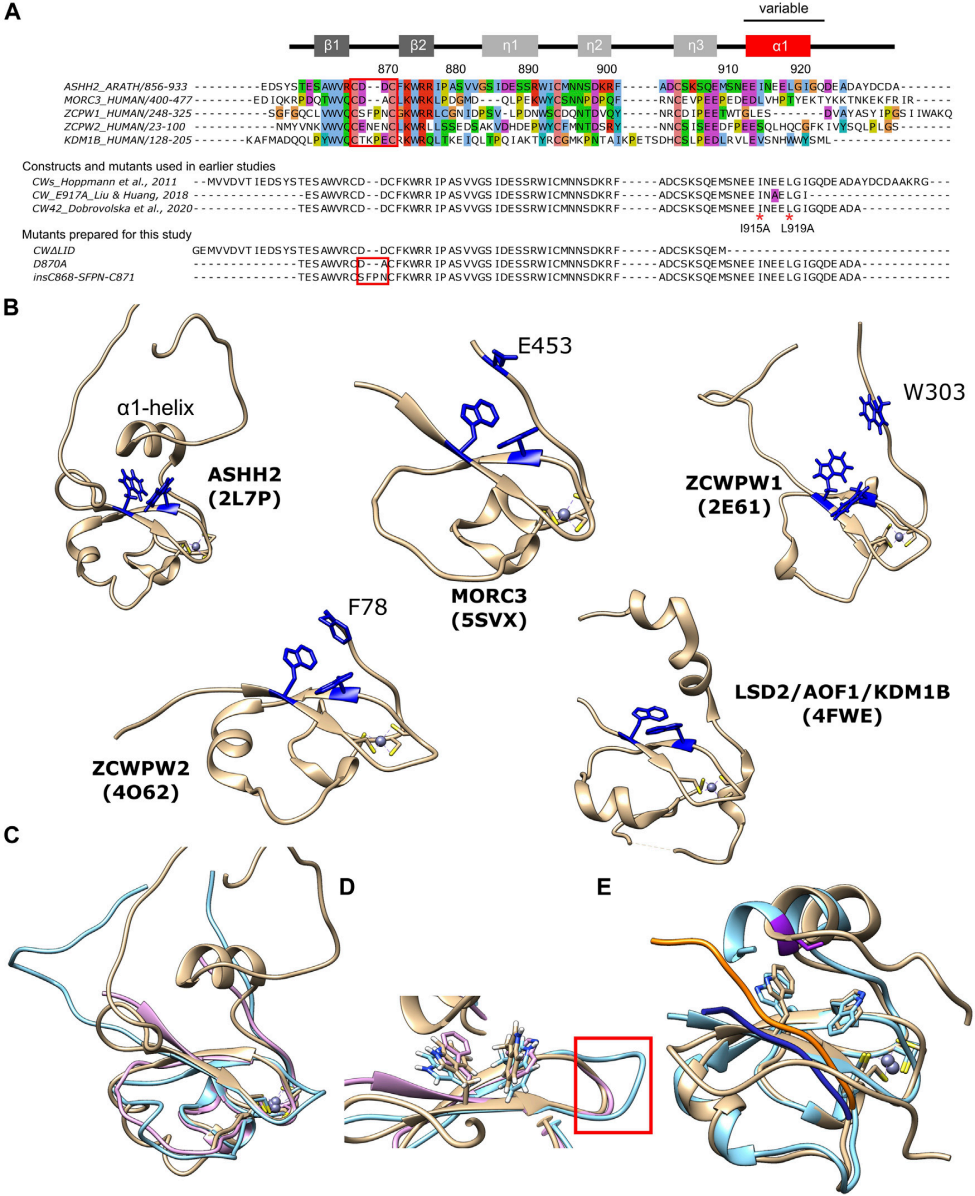
Overview of CW domains and structural analysis. **(A)** TOP: sequence alignment of CW domains of ASHH2 methyltransferase (*Arabidopsis thaliana*, Q2LAE1), MORC3 protein (*Homo sapiens*, Q14149), ZCWPW1 protein (*Homo sapiens*, Q9H0M4), ZCWPW2 protein (*Homo sapiens*, Q504Y3), and KDM1B histone demethylase (*Homo sapiens*, Q08EI0). The alignment was prepared using Jalview software and UniProt entries with ClustalO default parameters, and Clustalx coloring scheme was used. MIDDLE: CW domain constructs used in earlier studies on the ASHH2 CW domain by Hoppmann et al. (2011), Liu and Huang (2018), and Dobrovolska et al. (2020). The E917A mutation used by Liu and Huang (2018) is marked by purple color. I915A and L919A mutations were analyzed by Liu and Huang (2018) and in this work are marked by red * symbols. BOTTOM: sequences of the additional mutants (CW∆LID, D870A, and insC868-SFPN-C871) prepared exclusively for this work. The secondary structure of CW is indicated at the top of the panel. The red squares indicate the variable loop situated between the β-strands that form part of the binding site. These constructs also have an N-terminal sequence (“GSRRASVGSEF”) that is not shown in the figure; **(B)** overview of CW domain structures: ASHH2 (**2L7P**), MORC3 (**5SXV**), ZCWPW1 (**2E61**), ZCWPW2 (**4O62**), and LSD2/AOF1/KDM1B (**4FWE**) (tryptophans forming binding pockets and the variable C-terminal region residues, implicated in binding, are highlighted in blue); **(C)** superposition of CW domain structures: ASHH2 (brown), MORC3 (pink), and ZCWPW1 (blue); **(D)** close-up view of the binding pocket. The loops between the β-strands subject to the insC868-SFPN-C871 mutation are indicated by red square; and **(E)** overlay of CW structures in bound state solved by NMR (**6QXZ**, brown color, and the ligand is highlighted in orange) and X-ray crystallography (**5YVX**, blue color, and the ligand is highlighted in dark blue); purple color indicates E917A mutation on X-ray structure. Graphics were prepared using the UCSF Chimera software, and the pdb files represent domains in their unbound state (for B—D) and bound state (for E).

The regulation of gene expression and activity at the chromatin level relies on proteins that “read”, “write,” or “erase” post-translational modifications (PTMs) on histone tails and DNA. The proteins containing such domains are involved in genome organization and regulation by recognizing and modifying the PTM state of the genome compartments (DesJarlais and Tummino, 2016; Patel, 2016; Teske and Hadden, 2017). A key feature of histone tail recognition domains is that they recognize the presence of PTMs and have the ability to differentiate between their numbers and exact configurations (Taverna et al., 2007; Sanchez and Zhou, 2011; Mellor, 2006). The CW domain family is a histone tail methylation state reader shared among numerous organisms (vertebrates, vertebrate-infecting parasites, and higher plants). The name of the domain comes from its four conserved cysteines and three conserved tryptophans (Figure 1A). The conserved cysteines coordinate a Zn^2+^ ion essential for folding, and two of the three tryptophan residues form a π-cation-based binding pocket with high affinity toward methylated lysine residues found on the histone tails. The final tryptophan forms part of the hydrophobic core of the domain (Perry and Zhao, 2003; He et al., 2010; Hoppmann et al., 2011). The CW domain appears within large multidomain proteins, whose functions vary from protein family to protein family (Perry and Zhao, 2003). One example is the MORC family of ATPase chromatin remodelers. Here, the CW domain recruits proteins to the chromatin by recognizing H3K4me2/3 modifications. CW also regulates the ATPase activity of MORC3 by suspending its autoinhibition after binding to methylated H3 histone tails (Andrews et al., 2016; Li et al., 2016; Zhang et al., 2019). The MORC4 protein, despite having high structural and sequence similarity to MORC3, is not an autoinhibiting enzyme. For MORC4 activation, CW needs to interact with DNA in addition to binding H3K4me3 (Tencer et al., 2020). Another set of CW-containing proteins comprise the ZCWPW1 and ZCWPW2 PWWP domain proteins. Within these proteins, the CW function is unclear, but they recognize H4K20 methylation marks in addition to the H3K4me3 specificity conferred by the CW domain (He et al., 2010; Wang et al., 2009; Liu et al., 2016). In the transcriptional corepressor LSD2/AOF1/KDM1B that demethylates mono- and dimethyl H3K4 marks, CW appears to be inactive due to steric inaccessibility. However, CW contributes to the overall structural stability of the protein and regulates the enzyme’s activity and association with mitotic chromosomes (Shi et al., 2004; Yang et al., 2010; Zhang et al., 2013; Liu et al., 2016). In the MBD protein family, the ZmMBD101 protein maintains the repressed state of the *Mutator* genes, protecting plant genomes from mutagenesis caused by transposons. The role of the CW domain in this context is still unclear (Questa et al., 2016). CW also appears in ASHH2 (other names are SDG8 and EFS), a methyltransferase found in the small flowering plant *Arabidopsis thaliana*. ASHH2 is involved in the regulation of gene expression by histone H3 trimethylation at Lys-36 (H3K36me3). Within ASHH2, the CW domain preferentially binds to the H3K4me1 mark and presumably helps in docking the catalytic SET domain correctly onto the histone (Dong et al., 2008; Xu et al., 2008; Grini et al., 2009; Hoppmann et al., 2011).

The CW domain paralogs have different affinities toward different methylation states of H3K4. While the ASHH2 CW domain has higher affinity toward H3K4me1 (Hoppmann et al., 2011; Liu and Huang, 2018), the rest of the known CW domains bind stronger to H3K4me2 and H3K4me3 modifications (He et al., 2010; Andrews et al., 2016; Li et al., 2016; Liu et al., 2016). Previously, factors determining the ligand methylation state specificity of CW domains have been presented and discussed (Hoppmann et al., 2011; Liu and Huang, 2018; Dobrovolska et al., 2020). The C-terminal regions of the CW domain, due to their variability between paralogs, were suggested to be involved in ligand specificity. For example, the CW domain of ZCWPW1 is unique in that it has a non-conserved tryptophan residue (Trp303) at its C-terminal end (Figure 1B), and this tryptophan finalizes the binding pocket upon binding. The mutation of this tryptophan leads to reduced affinity (He et al., 2010). Its homolog ZCWPW2 has a phenylalanine residue (Phe78) in this position which also completes the binding pocket and might contribute to the selectivity of the methylation state (Figure 1B) (Liu et al., 2016). The CW domain of MORC3 proteins, in contrast, has Glu453 residue (Figure 1B), which finalizes the binding pocket and facilitates binding to methylated H3K4 peptides. Despite having the highest affinity towards H3K4me3 modification, its ability to differentiate between methylation states is reduced relative to what is observed for other CW domains (Andrews et al., 2016; Li et al., 2016; Liu et al., 2016). The ASHH2 subtype possesses a unique C-terminal α1-helix located right above the tryptophan-binding pocket (Figure 1B). Although not conserved, this helix is also part of the binding pocket (Liu and Huang, 2018; Dobrovolska et al., 2020), as the removal of this element leads to loss of ligand-binding ability (Hoppmann et al., 2011).

Recent X-ray (Liu *et al.*, PDB code: **5YVX**) and NMR (Dobrovolska *et al.*, PDB code: **6QXZ**) structural studies described the CW of ASHH2 in complex with H3K4me1. The published structures agreed on the core of the complex; however, the structural data related to the α1-helix and the following C-terminal regions differ (Figure 1E) (Liu and Huang, 2018; Dobrovolska et al., 2020). The studies also concluded differently with respect to binding mechanisms and determinants. The key residues for binding proposed by Liu *et al.* were L915, N916, and I919, residing on the α1-helix. These were proposed based on the crystal structure of the CW-H3K4me1 complex, where the domain construct ended right after the α1-helix (residue I921) and contained a mutation necessary for crystallization (E917A) close to the residues they identify as crucial for binding (Figure 1A). Dobrovolska *et al.* argued that the N916 residue is not a part of the interaction mechanism, as the NMR structure of the complex showed that this residue is oriented towards the solvent. The studies agreed, however, that L915 and I919 locked the methylated lysine of the ligand inside the binding pocket. The construct used by Dobrovolska *et al.* to solve the structure was longer at the C-terminal than the one used in the Liu *et al.’s* study, which allowed the elucidation of the role of the I921-Q923 region in binding. Analysis of the domain’s dynamics and flexibility using MD simulations and NMR further indicated that the binding mode of CW is described best by a conformational selection model (Dobrovolska et al., 2020). A conformational selection mechanism requires that the protein has a fluctuating structural ensemble that includes the conformation(s) required for binding, even in the absence of a ligand (Tsai et al., 1999; Csermely et al., 2010). Point mutations located in coils or elements influencing conformational equilibriums could disturb the interaction mechanism that depends on fine-tuned equilibriums.

The aforementioned works provide an understanding of the binding mechanism of the CW domain, but the question of selectivity still remains uncertain. Conformational selection requires that the correct binder (i.e., H3K4me1) stabilizes the bound conformation, whereas the other methylation states do not, or to a lesser degree. The presence of unstructured coils, loops, and mobile elements, and also the equilibrium positioning of the α1-helix unique to ASHH2 CW, might play a role here. Thus, the objective of this work was to investigate the possible coupling between these features and the fold stability as the selectivity determinants that allow the ASHH2 CW domain preferentially complex with H3K4me1. To achieve this goal, we prepared two mutants affecting the positioning of the tryptophans of the binding site and two mutants involved in positioning the α1-helix in the ligand-binding site. To assess the fundamental importance of this helix, we also prepared a deletion mutant, removing it entirely. We determined the affinities of H3K4me1/2/3 interacting with these mutants using ITC, performed thermal stability studies in the presence and absence of histone H3 mimicking peptides, and assessed changes to their fold using NMR fingerprinting.

## Results

### ASHH2 CW Domain Interaction With H3-Mimicking Peptides

#### Fold Assessment and Chemical Shift Perturbation Analysis

**FIGURE 2:**
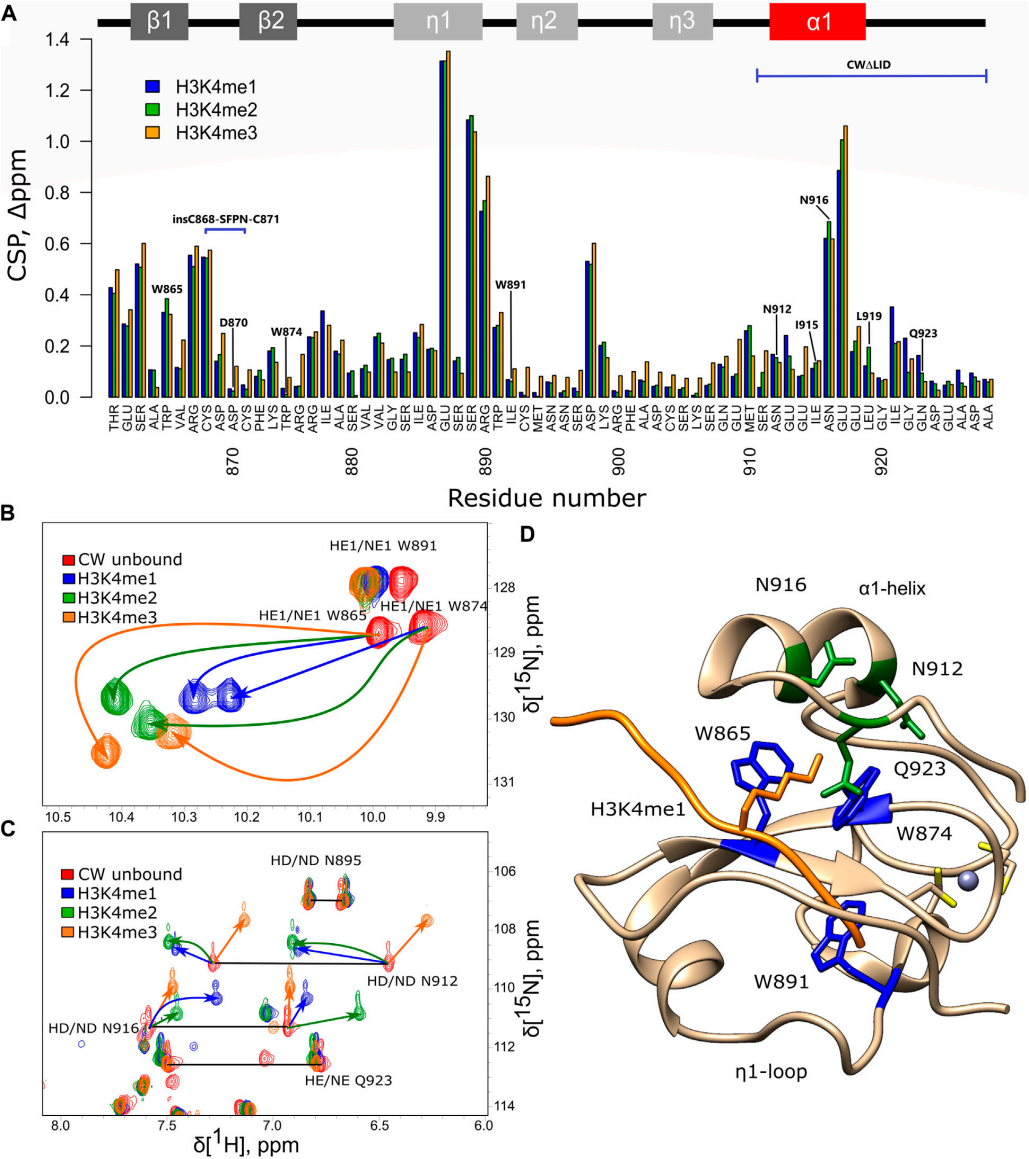
NMR analysis of CW interacting with histone-mimicking peptides. **(A)** Chemical shift perturbation, calculated for CW bound to the corresponding peptides (H3K4me1 blue, H3K4me2 green, and H3K4me3 orange). Secondary structure of CW is indicated at the top of the panel; **(B,C)** chemical shift of tryptophan, asparagine, and glutamine side-chain signals under binding of H3K4me1 (blue), H3K4me2 (green), and H3K4me3 (orange) peptides (red color—CW domain in unbound state); horizontal black lines connect signal pairs for ^15^Nɛ and ^15^Nδ signals of glutamines and aspargines, respectively; and **(D)** side-chain tryptophans (highlighted in blue), asparagines, and glutamine (highlighted in green) mapped on the CW structure in bound to H3K4me1 peptide (orange color) conformation (PDB: **6QXZ**); grey sphere is the Zn^2+^ ion coordinated by cysteins (yellow). All samples were dissolved in NMR buffer (20 mM phosphate buffer pH 6.4, 50 mM NaCl, and 1 mM DTT) with 10% D_2_O at 200 µM concentration of CW. The CW-to-peptide ratios were 1:4 for H3K4me1, 1:6 for H3K4me2, and 1:10 for H3K4me3.

The ASHH2 CW domain binds H3K4me1, H3K4me2, and H3K4me3, with the highest affinity toward H3K4me1 (Hoppmann et al., 2011). It is not known, however, whether the binding of these peptides is essentially the same or whether they affect the CW’s fold differently. The ^1^H-^15^N HSQC NMR spectra of CW in complex with mono-, di-, and trimethylated ligands were acquired and compared to the unbound form. Using the backbone chemical shift assignments available for the four situations (Dobrovolska et al., 2018), the chemical shift perturbation (CSP) was calculated, and the residues involved in the corresponding complex formation were determined (Figure 2A). The NMR data in the case of all the three histone-mimicking peptides suggest that binding causes fairly extensive structural changes that were not limited to W865 and W874 of the binding pocket. However, the HSQC spectra confirm that the CW domain remains folded when complexed with each of the peptides (Supplementary Figure S1). This observation is in line with the flexible nature of ASHH2 CW and the proposed mechanism of conformational selection (Dobrovolska et al., 2020). The most notable changes in chemical shifts are found in the first β-strand, perhaps related to the nascent β-sheet augmentation discussed by Dobrovolska et al., (2020), in the conserved W891 that is not part of the binding pocket, in the ɑ1-helix, and the η1-loop (Figure 2A).

The CSP data for the H3K4me1 peptide show that E913 in the α1-helix and I921-Q923 in the C-terminal tail are involved in the binding. Earlier, it was shown that I921 and Q923 are two of the key residues establishing contacts with H3K4me1 and forming part of the final cage-like configuration around K4me1 (Dobrovolska et al., 2020). In the case of the interaction with H3K4me2 and H3K4me3, the CSP values for these residues are less prominent, suggesting that they might not only be involved in mediating the interaction with the ligand but also contribute to selectivity towards H3K4me1. Binding to the H3K4me2 peptide has its most significant effect on W865 in the binding pocket, L873, the η1-region, M910, N916, and L919 of the α1-helix. Binding to H3K4me3 affected the structure to a somewhat larger degree, as the combined chemical shift values are slightly higher for the majority of the amino acids, especially at the η2- and η3-loops (Figure 2A). Yet, overall, the CSP data do not show much difference in how the backbone was affected by the three different peptides. The perturbation analysis takes into account only the backbone amide correlations, which may be insufficient for the detection of all features of these interactions.

Amino acid side-chain signals, corresponding to the NH groups of the tryptophans and asparagines and not included in the CSP analysis mentioned above, experience large shifts as a result of ligand binding. Notably, in each case of the ligand methylation state, the same NH signals had different patterns of shifts (in magnitude and trajectory), indicating that the chemical environment around these groups was affected differently (Figure 2B). The shifts suggest a change in the configuration of the tryptophan side chains in the binding pocket to accommodate the additional methyl group(s) of the ligand. A similar observation for the signals corresponding to the side chains of N912 and N916 suggests that the α1-helix also experiences methylation-dependent shifts in its positions (Figures 2C,D).

#### Dynamic Properties of the CW Domain Bound to Histone-Mimicking Peptides

The HSQC fingerprinting, CSP analysis, and differences in the chemical shifts of the side chains of the tryptophans and asparagines together suggest there may be subtle differences in how the ASHH2 CW domain responds to different ligand methylation states. Such differences may not be readily detectable by assessing chemical shifts only, as some of them reside among fluctuations in the domain’s structure and its dynamic properties that contribute little to crosspeak positions. Our earlier work provided a full NMR dynamics characterization for CW in the unbound and H3K4me1-bound states (Dobrovolska et al., 2020). Here, we expand part of this analysis by accumulating and comparing the hetNOE data for H3K4me1/2/3 peptides.

**FIGURE 3:**
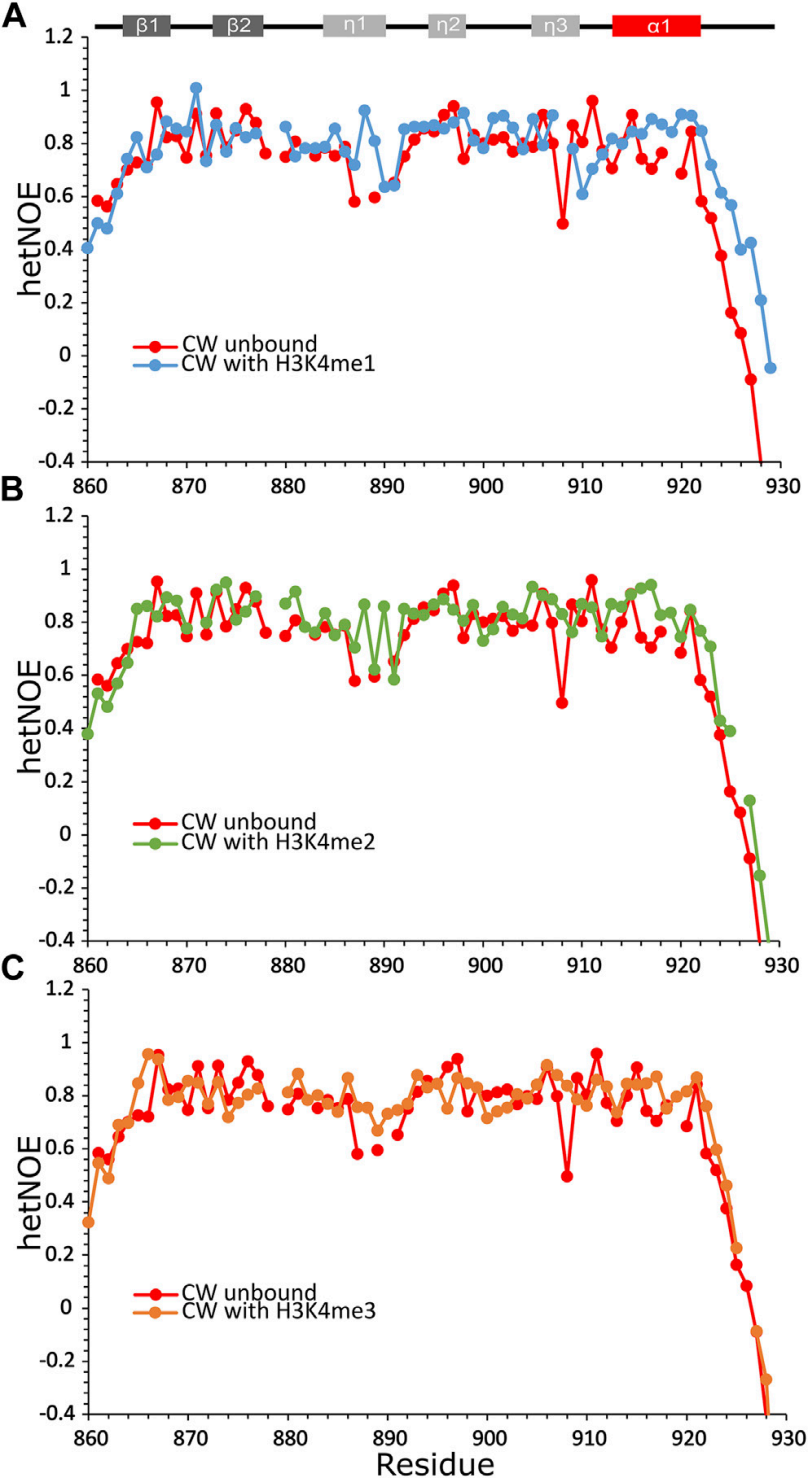
Heteronuclear ^1^H-^15^N NOEs data of CW bound to histone-mimicking peptides. **(A)** CW with H3K4me1 (blue circles); **(B)** CW with H3K4me2 peptide (green circles); and **(C)** CW with H3K4me3 peptide (orange circles). The data in each panel are overlaid with unbound CW (red circles). Secondary structure of CW is indicated at the top of the panels. All samples were dissolved in NMR buffer (20 mM phosphate buffer pH 6.4, 50 mM NaCl, and 1 mM DTT) with 10% D_2_O at 200 µM concentration of CW. The CW-to-peptide ratios were 1:4 for H3K4me1, 1:6 for H3K4me2, and 1:10 for H3K4me3.

Overall, the average hetNOE values for the domain bound to the peptides were around 0.8, indicating moderate local protein motions on the picosecond timescale (Kharchenko et al., 2020), except in the η1-loop, which remained quite flexible with hetNOE values as low as 0.5–0.7 (Figure 3). Generally, the binding of the H3K4me1 peptide results in movement restriction of the unstructured C-terminal I921-Q923 region. The η3-loop residue S907 shows high local mobility, as was reported in the work of Dobrovolska et al., (2020), with stabilization when the CW domain is complexed with H3K4me1/2/3 peptides. Binding to H3K4me3 has the least stabilizing effect on the α1-helix region and the η1-loop, and the overall dynamic behavior of CW looks more similar to that of the domain in its unbound state (Figure 3C). The results may indicate that the full shift in fold equilibrium occurs only with monomethylated ligands.

#### Thermodynamical Characterization of the Interaction

**FIGURE 4:**
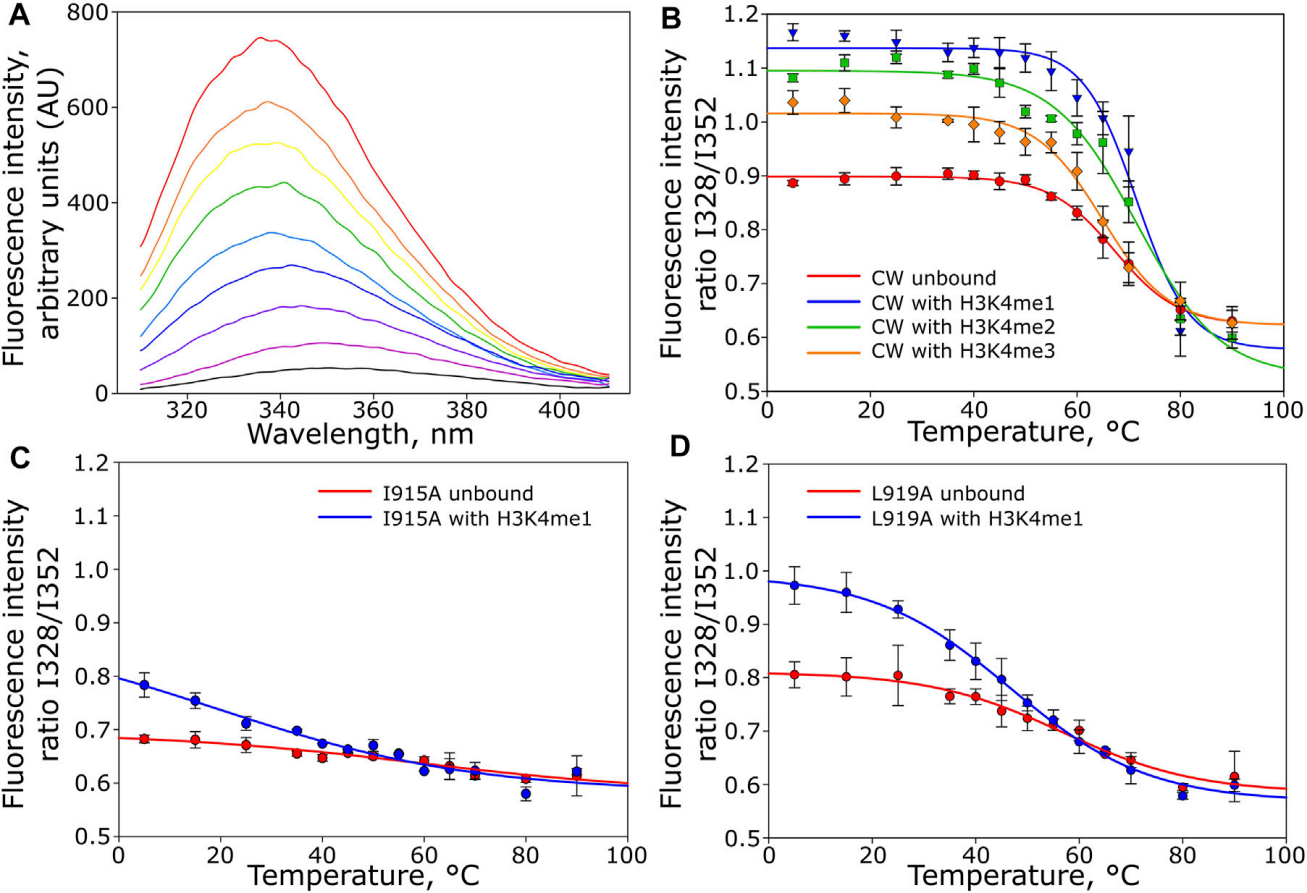
Thermal denaturation of CW monitor by intrinsic tryptophan fluorescence in unbound and bound states. **(A)** Representative fluorescence traces for wild-type CW bound to H3K4me1 (only selected traces are shown), rainbow colors indicate temperature increase from 5°C (black) to 90°C (red). **(B)** melting curves for the unbound CW domain (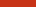) and CW domain in the presence of H3K4me1 (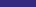), H3K4me2 (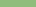), and H3K4me3 (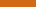) peptides; **(C)** melting curves for the unbound CW I915A mutant (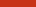) and in the presence of H3K4me1 peptide (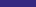); and **(D)** melting curves for the unbound CW L919A mutant (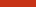) and in the presence of H3K4me1 peptide (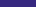). Number of replicates = 3.

Although primarily restricted to local movements on the picosecond timescale, the NMR analysis suggested that CW, when complexed with the peptides, samples available conformations differently at equilibrium. If a given methylation state fails to stabilize the complex, this should be reflected in its thermal stabilities. The CW domain in its unbound and bound states was therefore subjected to thermal denaturation analysis monitored by intrinsic tryptophan fluorescence. Complexation with H3K4me1 and H3K4me2 peptides increased the thermal stability of the domain: T_m_ = 67.3°C without peptide, T_m_ = 71.4°C with H3K4me1, and T_m_ = 71.6°C with H3K4me2. In contrast, interaction with H3K4me3 reduced this stability to T_m_ = 64.9°C (Figures 4A,B).

**FIGURE 5:**
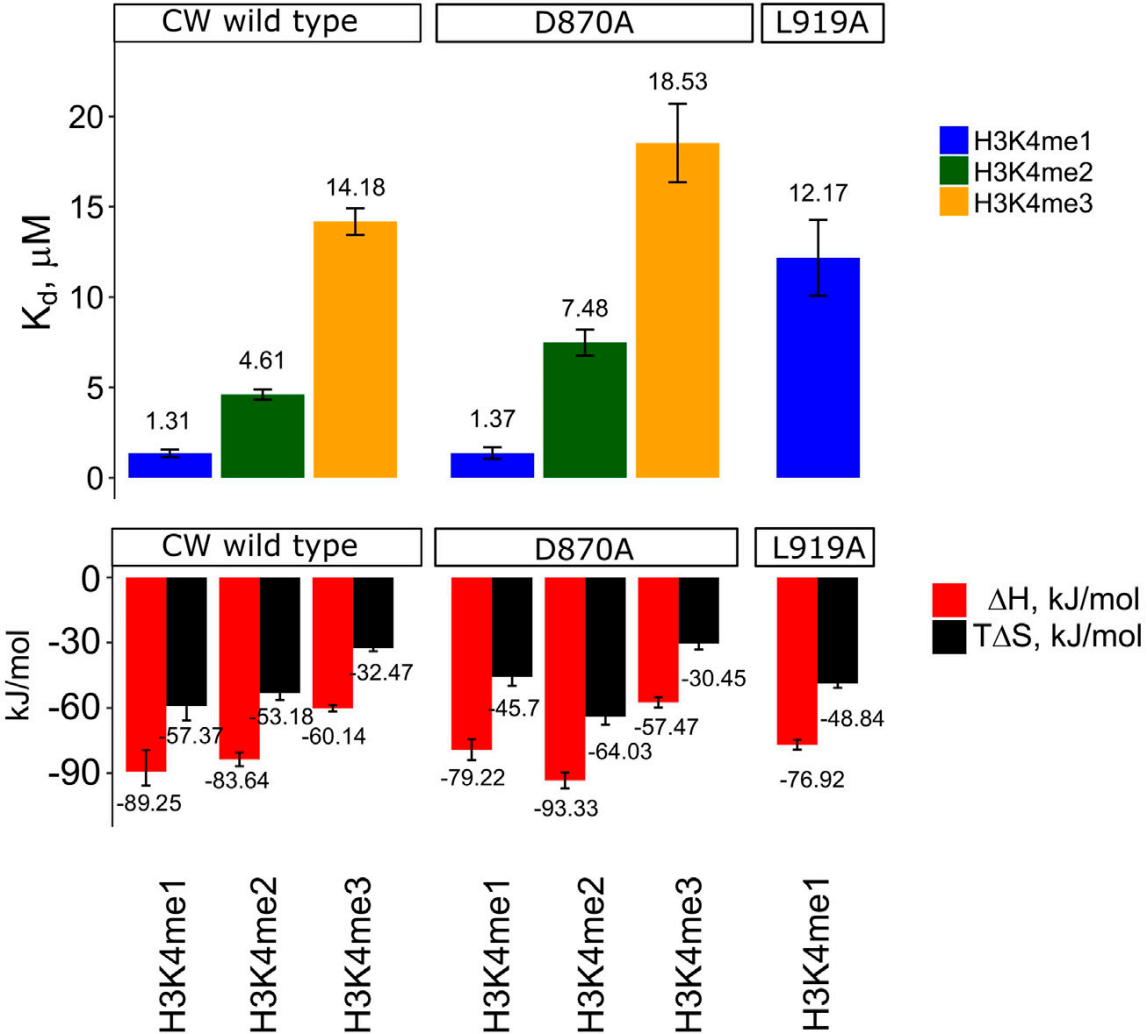
K_d_, ∆H, and T∆S values determined by ITC. Data shown only for WT CW and mutants D870A and L919A, as insC868-SFPN-C871 and I915A did not produce interpretable isotherms. Enthalpy of binding was determined by stepwise titration of 400–1,800 μM histone peptide to 50–180 μM sample dissolved in T7 buffer (25 mM Tris-HCL pH 7.0, 150 mM NaCl, and 1 mM TCEP) at 25°C. For the comparison, the entropic component was multiplied by the temperature. The values can be found in Supplementary Table S3. Error bars represent one standard deviation (n = 3). All pairwise comparisons between K_d_ values within the CW wild type group are significant (student's *t*-test, *p* < 0.01). D870A vs. CW wild type (me2/3), and L919A vs. CW wild type (me1), are significant (student's *t*-test, *p* < 0.05), while D870A vs. CW wild type (me1) is not (*p* = 0.416). Representative isotherms can be found in Supplementary Figures S2.

To study the thermodynamical forces underlying ligand binding and selectivity, we performed ITC measurements of CW interacting with H3K4me1/2/3 peptides. Interaction with the H3K4me1/2/3 peptides resulted in K_d_ = 1.3 ± 0.32 μM, K_d_ = 4.6 ± 0.28 μM, and K_d_ = 14.2 ± 0.74 μM, respectively. The binding enthalpies were similar (ΔH values of −89 ± 8.58 and −84 ± 3.11 kJ/mol) for H3K4me1 and H3K4me2 and lower (−60 ± 1.38 kJ/mol) for H3K4me3. Similarly, entropies associated with binding were ΔS = −192 ± 15.31 J/mol K, ΔS = −178 ± 10.82 J/mol K, and −109 ± 5.00 J/mol K, for H3K4me1, H3K4me2, and H3K4me3, respectively. Stoichiometric coefficients were in the range of 0.82–1.08. As a control, the unmodified H3K4 peptide was used and showed no binding. The results of the analysis are summarized in Figure 5 and Supplementary Table S3, and representative isotherms are shown in Supplementary Figures S2A-D.

#### Effect of α1-Helix Mutations on Binding Affinity, Domain Conformation, and Thermal Stability

**FIGURE 6:**
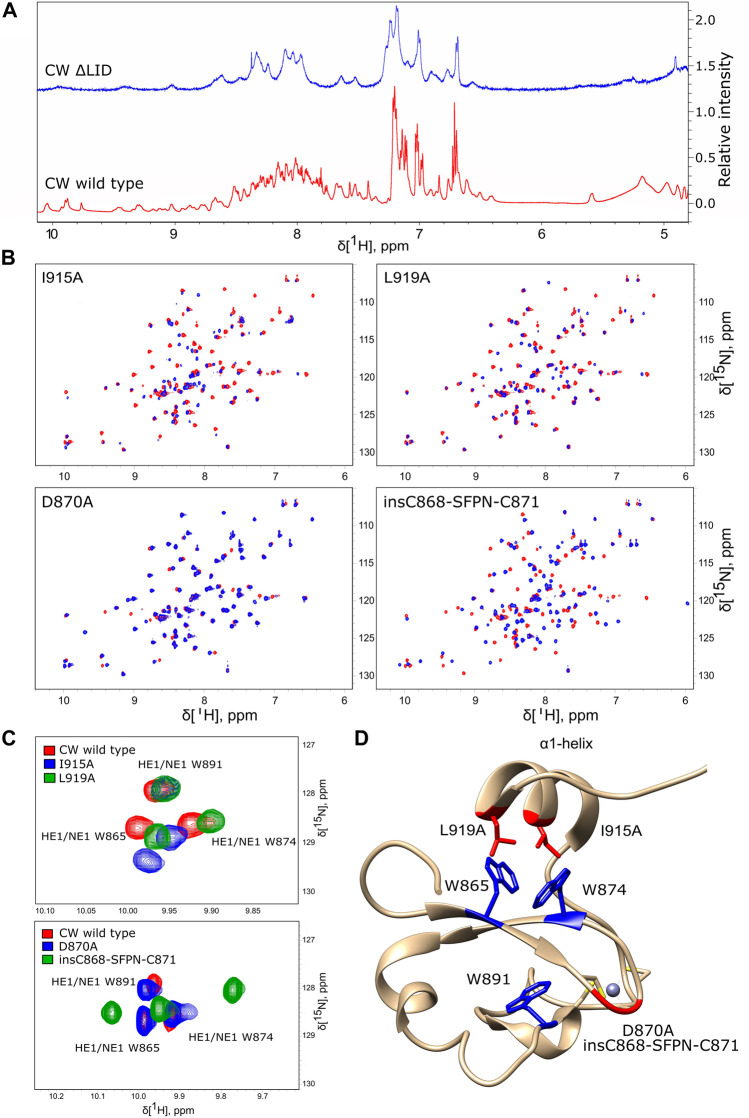
Mutation-induced structural disturbances analyzed by NMR. **(A)** Proton spectrum of CW ΔLID (blue) compared to the proton spectrum of wild-type CW (red). The CW ΔLID trace has been shifted up by 1.5 relative units to make comparison easier. **(B)** ^15^N HSQC spectra of I915A, L919A, D870A, and insC868-SFPN-C871 (blue color) overlaid with the spectra of WT CW (red color). **(C)** The effect of mutations in the α1-helix (red, WT CW; blue, I915A; and green, L919A (top panel)) and the effect of mutations in the loop between β-sheets (red, WT CW; blue, D870A; and green, insC868-SFPN-C871 (bottom panel)). **(D)** Location of the point mutations (red sticks) and insertion mutations (red backbone trace) mapped on the CW structure (PDB: **2L7P**). The tryptophans are highlighted in blue; grey sphere is the Zn^2+^ ion coordinated by cysteins (yellow).

Our previous study of CW binding to H3K4me1 showed that the α1-helix and the disordered region after it contribute critically to binding. A group of CW residues residing in this region established direct NOE contacts with the ligand and comprised L919, I915, and Q923 (Dobrovolska et al., 2020). Hoppmann *et al.* showed that, without this part, the protein is no longer capable of binding a ligand. It is, however, unclear whether this is due to loss of fold or the inability to retain the ligand in the otherwise intact binding site. To assess the impact the α1-helix has on the fold of the protein, the CWΔLID mutant (residues 911–928 removed, Figure 2A) was generated, and an ^1^H NMR spectrum was acquired. Compared to the wild type, the spectrum of the CWΔLID shows fewer and broader peaks spread across a narrower region, which indicates that the removal of the α1-helix leads to a fold disturbance (Figure 6A). This finding underscores the joint importance of both ligand binding and fold maintenance of the α1-helix and the C-terminal region.

To examine the role of the α1-helix in domain stability and specificity, we prepared I915A and L919A mutants. Their ^1^H-^15^N HSQC NMR spectra showed that the protein remains folded (Figures 6D,E). Compared to the wild type, I915A and L919A mutations showed an impact on the overall fingerprint, as more than 60% of the peaks were shifted substantially (more than 0.2 ppm) (Figure 6B). Thermal denaturation experiments showed that, compared to the native CW domain (T_m_ = 67.3°C), the L919A mutation significantly decreased the stability of the protein by 10.4°C (T_m_ = 56.9°C) (Figures 4C,D). Moreover, the binding of H3K4me1 decreased it further, by more than 10° in addition (T_m_ = 46.7°C). This behavior strikingly contrasts with the wild type where the ligand increased the T_m_ by ≈ 5°C. The I915 mutation had a more dramatic impact on the fold stability, as it was not possible to fit a curve to the data from the denaturation analysis, as the data do not display a sigmoidal model (Supplementary Figures S2I-K).

Changes in the binding preference for the I915A mutant were previously evaluated using ITC by Liu *et al.*; their conclusion linked it to a change in specificity from H3K4me1 to H3K4me3. When we performed the ITC experiment with the I915A mutant using our slightly longer construct (CW42), it was not possible to obtain an accurate K_d_ and other thermodynamic parameters, presumably because of its weak interaction with the peptides. The interaction of the L919A mutant with the H3K4me2 and H3K4me3 peptides could not be characterized either. In the case of H3K4me1 interaction with L919A, the K_d_, binding enthalpy, and entropy were determined to be 12.2 ± 2.10 μM, −77 ± 2.31 kJ/mol (ΔH), and −164 ± 6.61 J/mol K (ΔS), respectively. The stoichiometric coefficients were in the range of 0.86–0.88. The results are summarized in Figure 5 and Supplementary Table S3. The representative isotherms are shown in Supplementary Figures S2L-M. The shift in specificity reported by Liu *et al.* was not confirmed in our experiments (Liu and Huang, 2018).

### Binding Pocket Flexibility as Possible Determinant for Binding Preferences

Given the indications that dynamics play a role in binding and specificity, we are interested in the differences between bound and unbound equilibrium states. To compare *apo* and *holo* binding pocket geometries, we performed sequence and structural alignments of the CW domains from ASHH2 (2L7P), MORC3 (5SVX), and ZCWPW1 (2E61) (Figure 1A). The structural superimposition of ZCWPW1, ZCWPW2, MORC3 (humans and mice), and ASHH2 CW revealed different angles between the tryptophan side chains in the binding pocket (Figure 1D). ASHH2 has the narrowest angle (65°), which might partially explain its specificity towards H3K4me1. In the remaining structures, with a reported preference toward H3K4me3, the angle varies between 77° and 113°, where the lowest value corresponds to the binding of an unmethylated H3K4-ligand. On ligand binding, the angle increases, widening the binding pocket (results are summarized in Table 1). This expansion might be possible because of the flexibility of the loops surrounding the binding pocket, with ASHH2 having insufficient flexibility to open up the pocket for stable binding to the H3K4me3 ligand. The gap in ASHH2 and MORC3 sequence alignments (Figure 1A) translates into a shorter loop between two β-sheets, compared to ZCWPW1 (Figure 1D). Inside this loop, MORC3 has alanine in the place of ASHH2’s D870, and ZCWPW1 has the sequence S867-FP-N870 inserted relative to ASHH2. These variations in length and composition of the loop may impact the orientation of the β-strands and, thus, also on the positioning of the conserved tryptophans in the binding pocket.

**TABLE 1:** Angles between tryptophan side chains forming the binding pocket. Tryptophan angle measurements were performed manually with Screen Scales from Talon Designs LLP and automatically by fitting a plane through the coordinates of the carbon and nitrogen atoms in the two tryptophan side chains and solving the angle between the two planes. The Trp numbers refer to the residue numbering used in the corresponding PDB ID. If more than one chain is available in the structures, only chain A is used for this calculation.

UniProt ID	PDB ID	Trp number	Ligand	Angle, manual	Angle, automatic	Highest specificity	Reference
ASHH2_ARATH	2L7P	28, 37	Unbound	65.9	64.9	H3K4me1	Hoppmann et al. (2011), Liu and Huang (2018), Dobrovolska et al. (2020)
MORC3_HUMAN	6QXZ	865, 874	H3K4me1	94.4	94.4	H3K4me3	Andrews et al. (2016), Liu et al. (2016)
MORC3_MOUSE	5YVX	865, 874	H3K4me1	113.0	113.0	H3K4me3	Li et al. (2016)
ZCWPW1_HUMAN	4QQ4	410, 419	H3K4	99.6	100.0	H3K4me3	He et al. (2010)
ZCWPW2_HUMAN	5SVY	410, 419	H3K4me1	99.1	98.0	H3K4me3	Liu et al. (2016)
	5SVX	4, 13	H3K4me3	100.5	100.0		
	5IX2	410, 419	H3K4	78.0	76.8		
	5IX1	410, 419	H3K4me3	89.9	88.6		
	2E61	18, 29	Unbound	104.1	104.4		
	2RR4	256, 267	H3K4me3	107.6	105.6		
	4O62	30, 41	H3K4me3	113.2	111.2		

Loops have been implicated in the binding mechanism of the CW domain, where they regulate the positioning of the α1-helix (η3-loop and the post-helix C-terminal coil). Additionally, the η1-loop also interacts with the ligand on complex formation, and together, these unstructured elements mediate binding and stabilize the complex (Dobrovolska et al., 2020). To study the role of the loop that connects the two β-sheets scaffolding the binding pocket, the D870A and insC868-SFPN-C871 mutants were prepared, with mutations that correspond to the composition of the MORC3 and ZCWPW1 loops, respectively. The folded states of the mutants were verified by ^15^N HSQC fingerprinting (Figure 6B). The D870A mutant had little effect on the structure, affecting only around 7% of the signals in the fingerprint. The insC868-SFPN-C871 insertion mutant had a more pronounced effect on the structure, resulting in a shift of around 45% of signals (Figure 6B). Changes in the binding preferences of the D870A and insC868-SFPN-C871 mutants were evaluated by ITC.

It was not possible to obtain K_d_ and other thermodynamic parameters for the insC868-SFPN-C871 mutant because of weak interactions (Supplementary Figures S2H show the representative isotherm for interaction with H3K4me1). A change in selectivity was not observed, and interaction of the D870A sample with H3K4me1 was comparable to WT values (K_d_ = 1.4 ± 0.32 μM, binding enthalpy ΔH = −79 ± 4.81 kJ/mol, and entropy ΔS = −153 ± 13.75 J/mol K). This mutation gave a decreased binding affinity toward the H3K4me2 and me3 peptides; however, the interaction with H3K4me2 resulted in K_d_ = 7.5 ± 0.72 μM, ΔH = −93 ± 3.65 kJ/mol, and ΔS = −215 ± 12.06 J/mol K. For H3K4me3, these binding parameters were K_d_ = 18.5 ± 2.17 μM, ΔH = −57 ± 2.37 kJ/mol, and ΔS = −102 ± 8.90 J/mol K. The stoichiometric binding coefficients were in the range of 0.96–1.01. The results are summarized in Figure 5 and Supplementary Table S3 with representative isotherms shown in Supplementary Figures S2E-G.

### Effect of Mutations on the Environment of the Tryptophan Side Chains

Chemical shift analysis of the unbound state and of the H3K4me1-3 bound situations suggested that the side chains were more sensitive to the binding pocket environment compared to their respective backbone atoms. Therefore, we also compared the spectra of insC868-SFPN-C871, D870A, I915A, and L919A to the spectra of the WT with a focus on side-chain NH cross peaks. Signals from the ε-NH nuclei of the aromatic side chain of tryptophans are located in the ^1^H 7–11 ppm and ^15^N 128–130 ppm regions of the ^1^H-^15^N HSQC spectra. Mutations in the α1-helix did not affect the core tryptophan W891 but significantly affected the tryptophan side chains in the binding pocket (W865 and W874). For L919A, ^15^Nɛ resonances were shifted upfield, maintaining a similar pattern as in the wild-type domain. The I915A mutation affected the side chains somewhat differently, with the ^15^Nɛ resonance shifted downfield in the case of W865 (Figure 6C). The mutant with the MORC3-like loop (D870A) maintained a chemical environment similar to the WT situation, with a slight shift of the signal from the core tryptophan side chain (W891). The mutant with the ZCWPW1-like loop (insC868-SFPN-C871) had the most pronounced effect on the core tryptophan (W891) and also shifted the ^15^Nɛ resonances of the binding pocket’s tryptophan side chains upfield (W865 and W874) (Figure 6C).

## Discussion

Early characterization of ASHH2 CW domain binding behavior was performed by Hoppmann et al. (2011) and later expanded with the structures of the bound state by Liu and Huang (2018) and Dobrovolska et al. (2020). The latter study and ligand titration experiments indicated that the CW domain sampled a range of conformations across the ns–ms timescale at equilibrium (Kharchenko et al., 2020). Relaxation–dispersion experiments were then used to verify that some residues exhibited exchange on the timescales (i.e., up to ms) associated with conformational selection, a binding mode where ligands bind the correct conformation from a continuum of existing conformations (Farber and Mittermaier, 2015; Dobrovolska et al., 2020). Mutations of these residues (D886, S907, and Q908) interfered with the H3K4me1 binding, indicating that this behavior was indeed linked to function (Dobrovolska et al., 2020).

Ligand binding has different effects on the protein stability depending on the interaction system. Typically, a ligand stabilizes the structure, but in case of binding to partially unfolded intermediate states of proteins, the stability can be decreased (D'Auria et al., 2005; Matulis et al., 2005; Cimmperman et al., 2008; Gianni and Jemth, 2019). Intrinsically disordered proteins interacting with ligands are characterized by the formation of secondary structure elements on binding (Iešmantavičius et al., 2014; Schneider et al., 2015). This is, in turn, associated with a reduction in the dynamics of flexible loops, shrinking in protein hydrodynamic size and a reduction in conformational entropy (D'Auria et al., 2005; Zhu et al., 2019; Di Lella et al., 2010; Steiner et al., 2013). The affinity of the interaction arises from enthalpy–entropy compensations, where the most stable complex is formed with the most favorable thermodynamic terms (Ferrante and Gorski, 2012). CW is not an intrinsically disordered protein, but is rich in coils. In this study, we explored how the domain dynamics, thermodynamics binding parameters, and thermal stability changed as a function of the ligand methylation state with a focus on CW dynamic elements. We found that the interaction with H3K4me1 and H3K4me2 caused fold-stabilization whereas with H3K4me3 did not. We also found that removing or altering the α1-helix through mutations had large effects on the domain fold. Furthermore, mutations in the loop structures not explored previously had a marked effect on its thermal stability, fold, and the domain’s ability to interact with H3K4me1/2/3 histone mimics.

The ITC data show that peptide binding is enthalpy driven, and the difference in affinity arises from different enthalpy–entropy contributions (Homans and Peters, 2007; Ladbury, 2010). H3K4me1 shows the most favorable enthalpy contribution of the three histone-mimicking peptides. Interaction with the other peptides showed a smaller enthalpy contribution. As the change in enthalpy in non-strict terms characterizes the number of non-covalent bonds formed on complexation (Du et al., 2016), this can be interpreted as the formation of fewer non-covalent interaction contacts for H3K4me2 and -me3 cases. Interaction with these peptides is also characterized by an increase in entropy terms when compared to H3K4me1 interaction. The results show that the dominant driving force is the enthalpy overcoming an unfavorable entropic term, which is consistent with the conformational selection mechanism, as lock-and-key type interactions are driven by the solvent gain in entropy (Li et al., 2014; Corbett et al., 2005; Du et al., 2016). The K_d_ values for the CW interaction obtained using ITC do not match entirely with the values obtained by Hoppmann *et al.* for H3K4me2 (K_d_ of 2.1 vs. 4.61 µM in the present study) and H3K4me3 (K_d_ of 4 vs. 14.18 µM in the present study), but are comparable in the case of H3K4me1. Compared to Liu *et al.*’s data, the values reported are in better agreement. The discrepancy may be explained by differences in the methods and protein constructs used. Hoppmann *et al.* employed SPR, which is sensitive to mass transfer, re-binding, and surface effects (Berggård et al., 2007; Schuck and Zhao, 2010). In addition, the study used a GST-ASHH2-CW construct, where the presence of the GST-tag could potentially affect the interaction (Nguyen et al., 2015). However, both studies concluded that H3K4me1 is the strongest binder, whereas H3K4me3 is the weakest.

The thermal denaturation analysis showed a reduction in the stability of the CW-H3K4me3 complex, which matches an unfavorable shift in the enthalpy–entropy contribution of the interaction and a somewhat greater fold perturbance as seen in the ITC and NMR analyses, respectively. The trimethylated side chain of the H3K4 lysine is bulkier compared to the di- and monomethylated situations. Insertion of the larger residue in the binding pocket seems to expand the binding pocket, which, to a greater extent, perturbes the fold. In the available NMR structure of the bound state, the H3K4me1 side chain is tightly surrounded by the C-terminal residues in the bound situation. On selection of the right conformation, L919, I921, and Q923 form a tight complementary fit together with the two binding pocket tryptophans, W865 and W874 (Dobrovolska et al., 2020). This consolidation cannot be achieved for H3K4me3. It would seem that such a complex might disrupt the network of interactions between the mobile α1-helix, the C-terminal tail, and the tryptophans lining the binding pocket that consolidates in the case of H3K4me1/2 binding. This is consistent with the hetNOE data, which showed that the structure of CW is adjusted to the favorably bound form by restricting the mobility of the flexible η1- and η3-loops and the I921-Q923 unstructured region in the C-terminal region. This region remains mobile when CW is bound to H3K4me3, possibly related to the increase in entropy observed in the thermodynamic binding parameters of this ligand. Taken together, these results suggest that the η1- and η3-loops and the highly mobile C-terminal region of CW are able to make stable contacts only when complexed with the H3K4me1 peptide. This analysis is in general agreement with the model presented by Liu *et al.*, where steric hindrances between the binding pocket and trimethylated group were discussed. However, our discussion is not restricted to the immediate binding site, and we arrive at these conclusions primarily considering highly mobile elements of the domain.

Compared to other known structures of CW domains, the ASHH2 subtype has the α1-helix, whose function seems to be related to the specificity of the domain (Liu and Huang, 2018). Our data suggest that truncation of the helix eliminates binding by disturbing the domain’s fold. Point mutations of the amino acids whose side chains are oriented toward the tryptophan binding pocket confirmed that the structure is stabilized by these residues. Comparing the I915A and L919A mutations, we conclude that I915 contributes the most to structural stabilization and is also crucial for maintaining the fold optimal for binding. The I915A mutation had a pronounced effect on the overall structure and chemical environment around the tryptophan side chains of the binding pocket and, thus, likely affected its geometry, which resulted in the loss of quantifiable interaction. It was not possible to determine the T_m_ for the I915A mutant with intrinsic tryptophan fluorescence spectroscopy, as the data could not be fitted by a sigmoidal curve. Knowing from HSQC fingerprinting that the I915A mutant remains folded, such behavior may be explained by the exposure of tryptophans to the environment (Duy and Fitter, 2006). One possibility is that the I915A mutation could distort or displace the α1-helix exposing the tryptophan side chains, as also seems from the change in the chemical environment around indole rings of the tryptophans in the HSQC side-chain analysis. The L919A mutation showed decreased affinity to H3K4me1 arising from changes in the balance between enthalpy and entropy contributions of the interaction. Higher values for enthalpy and entropy indicate that the binding of the peptide was not complete relative to the native situation, and thermal stability measurements indicated a notable destabilization of the complex. These observations are also in agreement with the conclusions from the work of Liu *et al.*, where they demonstrated the importance of I915 and L919 in the formation of the binding pocket. However, their ITC results and conclusion about selectivity of the domain have to be treated with care, as their reported stoichiometric coefficient values did not approach 1:1 binding, as would be expected for a well-optimized experimental system (Perozzo et al., 2004).

Working with CW has highlighted the importance of coils and flexible elements in its binding action. The MORC3-like loop mutant (D870A) and the ZCWPW1-like loop (insC868-SFPN-C871) were explored to test the indirect effect alterations these loops might have on the β-sheet scaffolding of the tryptophans of the binding pocket. The D870A mutation did not affect the chemical environment of the tryptophans in the binding pocket, in line with the observation that the interaction of the D870A mutant with histone-mimicking peptides was similar to the wild-type situation. The interaction is driven by enthalpy, but with slightly reduced affinities toward H3K4me2 and H3K4me3. In the case of the insC868-SFPN-C871 mutant, however, the interaction was significantly weakened, and it was not possible to find the biding isotherm by ITC. This loss in affinity can be explained by a high degree of structural perturbance of the domain, as indicated by its HSQC fingerprint.

There is unfortunately little structural information available on how the CW domain might act in the context of the full ASHH2 protein. Its UNIPROT alpha-fold prediction (entry: Q2LAE1) is rich in low-confidence regions, including the interesting C-terminal region and its extension toward its catalytic domain. It is possible that selectivity toward histones is increased in the full context of the enzyme, or that the CW domain elicits changes to the domain organization of the full protein when bound. The work carried out by Dong *et al.* and Xu *et al.* provides some interesting findings regarding this (Dong et al., 2008; Xu et al., 2008). These groups performed methyltransferase assays with a radiography-labeled methyl group donor. Xu *et al.* showed that a truncated construct of ASHH2 lacking the CW domain and retaining only its catalytically relevant methyltransferase domains is able to methylate *in vitro* not only H3 histone but also H4. Dong *et al.* in their study used a full-length ASHH2, which was shown to “write” a methylation mark on H3 histone only. These observations together immediately suggest that the CW domain of ASHH2 methyltransferase might function by restricting the SET domain activity specifically toward H3 histones.

## Materials and Methods

### Materials

The histone H3 tail-mimicking peptides were synthesized by Lifetein (sequences are listed in Supplementary Table S2) and had 95% purity (assessed by mass spectrometry). Buffer components and chemicals were purchased from Sigma-Aldrich. D_2_O, ^15^N-enriched (99%) NH_4_Cl, and ^13^C-enriched (99%) glucose were purchased from Cambridge Isotope Laboratories, Inc. (Tewksbury, MA, United States), and SVCP-Super-3-103.5 NMR tubes were acquired from Norell Inc. (Morganton, NC, United States).

### Analysis of Known Structures

To determine the angles between the tryptophan residues in different CW domains, a multiple-structure alignment was performed on the A chain of known CW domain structures using the POSA web tool (Ye and Godzik, 2004). The results were visualized using UCSF Chimera (Pettersen et al., 2004) and PyMOL, and the measurements of the angles between the two tryptophan residues were made manually with Screen Scales from Talon Designs LLP and automatically by fitting a plane through the coordinates of the atoms in the two tryptophan side chains and solving the angle between the two planes. The Git code is available at https://github.com/oodegard/CW_domain_paper. Sequence alignment was performed using Jalview software (Waterhouse et al., 2009) with Clustal Ω algorithm with default parameters.

### Protein Expression and Purification

Protein expression and purification was performed as described in the work of Dobrovolska et al. (2018). Mutant versions of the ASHH2 CW domain were generated using site-directed mutagenesis and PCR. The primers used are listed in Supplementary Table S1. After PCR, the reaction mix was treated with DpnI, and linearized DNA was ligated at room temperature for 20 min in a reaction containing PCR product, NEB4 buffer, T4 ligase, T4 polynucleotide kinase, 1 mM ATP, and 10 mM DTT. Mutant CW constructs were expressed and purified as described previously. All the constructs were verified by sequencing.

### Isothermal Titration Calorimetry

For all measurements, the temperature and stirring rate were kept constant at 25°C and 300 rpm, respectively. The sample concentration was 50–180 μM, and the enthalpy of binding was determined by the stepwise titration of 400–1,800 μM histone peptide. Each CW sample (wt and mutants) was analyzed with H3K4me1, H3K4me2, and H3K4me3 (peptide sequences listed in Supplementary Table S2). For each experiment, 2 μl of peptide was injected 22 times with 300 s intervals between injections. The experiments were performed in triplicates. Both proteins and peptides were dissolved in T7 buffer (25 mM Tris-HCL pH 7.0, 150 mM NaCl, and 1 mM TCEP), and the heat of peptide dilution into T7 buffer was subtracted from the measurement. The binding parameters were determined from the integrated peak areas with independent modelling, using the TA Instruments NanoAnalyze V 2.4.1 software. Pairwise Student's *T*-test where applied (*n* = 3) in selected instances to test for significance, with threshold valiues set to *P* = 0.05.

### Thermal Denaturation

The melting point of the different CW domain constructs, defined as the temperature at the inflection point of I328/I352 during heat denaturation, was determined with and without the ligand. Each sample was subjected to a temperature gradient ranging from 5 to 90°C. Each 5–10°C increase in temperature was allowed to come to thermal equilibrium before measurements were taken. Fluorescence was recorded in the range between 310 and 410 nm with a scanning rate of 100 nm/min, two scans per sample. Each sample was analyzed in triplicates. The cuvette was equipped with a lid to prevent evaporation from the sample. The samples were dissolved in T7 buffer, the protein concentration was 10–20 μM, and the ratio of protein to peptide was 1:6. The corresponding blanks (buffer or peptide solution) were subtracted from the measurements. The ratio of fluorescence intensities at 328 and 352 nm was plotted against temperature. A sigmoidal curve with four variables (Eq. 1) was fitted between parallels to obtain the inflection point (taken as the melting temperature, T_m_). Curve fitting was performed with SigmaPlot v13.0 followed by a one-way ANOVA statistical test with a *p* value threshold of 0.05.

$$f\left(x\right)=y_{0}+\frac{a}{1+e^{(-b(x-x_{0}))}}\;.$$ (1)

Here, *f(x)* is a function of temperature, *y*_*0*_ is the slope of the pre-transition state, *a* is the slope of the post-transition state, *x*_*0*_ is the infection point of the curve, and *b* is the slope of the transition state at the inflection point *x*_*0*_.

### NMR Spectroscopy

The protein samples were dissolved in NMR buffer (20 mM phosphate buffer pH 6.4, 50 mM NaCl, and 1 mM DTT) with 10% D_2_O at 200 µM concentration of CW. The CW-to-peptide ratios were 1:4 for H3K4me1, 1:6 for H3K4me2, and 1:10 for H3K4me3. All NMR data were acquired on a Bruker Ascend 850 MHz instrument, fitted with a cryogenically cooled TCI probe, and at 300 K. The data were processed in TopSpin 3.5 pl 6.2.^1^H-^15^N-HSQC fingerprints were acquired using the f3 channel, ^15^N decoupling during acquisition, water flip-back pulse, and echo/anti-echo-TPPI gradient selection (Schleucher et al., 1994). The typical parameters were p1 7.06 µs, d1 1.0 s, SW 15.92 ppm (F2) and 35 ppm (F1), o1p 4.7 ppm, and o3p 35 ppm. TD was set to 2048 and 128 in F2 and F1, respectively. The data were acquired using 50% non-uniform sampling. For processing, a forward complex linear prediction was applied in F1 up to 256 points and zero-filled up to 512 points. The FIDs were apodized using a squared cosine function in both dimensions before Fourier transformation. HNCA, HNcoCA, CBCAcoNH, and CBCANH spectra were acquired for the purpose of assignment verification, based on BioMagRes entries 27250 and 27251. All the experiments were acquired using time-optimized NMR (Lescop et al., 2007) and were set up using the standard parameter files provided by the software of the instrument provider. Typical acquisition and processing parameters were as mentioned previously, except that TD for the ^1^H-dimension was 698 and SW was 14.00 ppm. In addition, the TD for the ^13^C-dimension was (where applicable) 64 points with a linear prediction up to 80. The NUS-amount for the 3D experiments was set to 25%. The processed NMR data were imported into CARA (Keller, 2005), for assignment and further analysis. Assigned HSQC spectra for wild-type CW in the unbound state and bound to corresponding peptides were used to calculate chemical shift perturbations with Eq. 2 and a scaling factor, α_N_, of 0.17 (Tochio et al., 2000; Piserchio et al., 2002).

$$\Delta ppm=\sqrt{{\left(\Delta \delta_{HN}\right)}^{2}+{\left(\Delta \delta_{N}*\alpha_{N}\right)}^{2}}.$$ (2)

Here, *Δppm* is the combined chemical shift; Δ*δ*_*HN*_ is the amide proton chemical shift, ppm; Δ*δ*_*N*_ is the nitrogen chemical shift, ppm; and *α*_*N*_ is the scaling factor.

### Dynamics Measurements

All the samples were dissolved in the same NMR buffer (20 mM phosphate buffer pH 6.4, 50 mM NaCl, and 1 mM DTT) with 10% D_2_O at 200 µM concentration of CW, with the same CW-to-peptide ratios: 1:4 for H3K4me1, 1:6 for H3K4me2, and 1:10 for H3K4me3. Dynamics measurements were performed as described in the work of Dobrovolska et al. (2020). In brief, for the determination of heteronuclear NOEs, two ^1^H-^15^N HSQC datasets were recorded at 300 K. A recycling delay of 3 s was used between transients. Relaxation delays of 20, 60, 80, 100, 200, 400, 600, 800, 1,000, 1,200, and 1,400 ms were recorded for T1 measurements, and relaxation delays of 16, 30, 60, 95, 125, 160, 190, 220, 250, 345, 440, and 500 ms were recorded for T2 measurements. The heteronuclear NOE values were calculated as the ratio of the steady-state intensities measured with and without the saturation of the proton magnetization. The data were processed and analyzed using NMRPipe (Delaglio et al., 1995) on NMRBox (Maciejewski et al., 2017).

## Data Availability

NMR assignments are available, and can be found in the BioMagRes Data bank: https://bmrb.io/. Accession numbers are 27250 and 27251, for the free and H3K4me1-bound situation, respectively.

## Abbreviations

PTM, post-translational modification; H3K4meX, histone tail peptide methylated 0–3 times at position lysine 4; H3K36me3, histone H3 trimethylation at lysine 36; hetNOE, heteronuclear nuclear Overhauser effect; ITC, isothermal titration calorimetry; NMR, nuclear magnetic resonance; NOE, nuclear Overhauser effect; HSQC, heteronuclear single quantum coherence; and CSP, chemical shift perturbation.

## References

[B1] Andrews F. H. Tong Q. Sullivan K. D. Cornett E. M. Zhang Y. Ali M. (2016). Multivalent Chromatin Engagement and Inter-Domain Crosstalk Regulate MORC3 ATPase. Cel Rep. 16, 3195–3207. 10.1016/j.celrep.2016.08.050 PMC5074691 27653685

[B2] Berggård T. Linse S. James P. (2007). Methods for the Detection and Analysis of Protein-Protein Interactions. Proteomics 7, 2833–2842. 10.1002/pmic.200700131 17640003

[B3] Cimmperman P. Baranauskienė L. Jachimovičiūtė S. Jachno J. Torresan J. Michailovienė V. (2008). A Quantitative Model of thermal Stabilization and Destabilization of Proteins by Ligands. Biophysical J. 95, 3222–3231. 10.1529/biophysj.108.134973 PMC2547457 18599640

[B4] Corbett P. T. Tong L. H. Sanders J. K. M. Otto S. (2005). Diastereoselective Amplification of an Induced-Fit Receptor from a Dynamic Combinatorial Library. J. Am. Chem. Soc. 127, 8902–8903. 10.1021/ja050790i 15969538

[B5] Csermely P. Palotai R. Nussinov R. (2010). Induced Fit, Conformational Selection and Independent Dynamic Segments: An Extended View of Binding Events. Trends Biochem. Sci. 35, 539–546. 10.1016/j.tibs.2010.04.009 20541943 PMC3018770

[B6] D'Auria S. Scirè A. Varriale A. Scognamiglio V. Staiano M. Ausili A. (2005). Binding of Glutamine to Glutamine-Binding Protein from *Escherichia C* Induces Changes in Protein Structure and Increases Protein Stability. Proteins 58, 80–87. 10.1002/prot.20289 15517590

[B7] Delaglio F. Grzesiek S. Vuister G. W. Zhu G. Pfeifer J. Bax A. (1995). NMRPipe: A Multidimensional Spectral Processing System Based on UNIX Pipes. J. Biomol. NMR 6, 277–293. 10.1007/BF00197809 8520220

[B8] DesJarlais R. Tummino P. J. (2016). Role of Histone-Modifying Enzymes and Their Complexes in Regulation of Chromatin Biology. Biochemistry 55, 1584–1599. 10.1021/acs.biochem.5b01210 26745824

[B9] Di Lella S. Martí M. A. Croci D. O. Guardia C. M. A. Díaz-Ricci J. C. Rabinovich G. A. (2010). Linking the Structure and Thermal Stability of β-Galactoside-Binding Protein Galectin-1 to Ligand Binding and Dimerization Equilibria. Biochemistry 49, 7652–7658. 10.1021/bi100356g 20666428

[B10] Dobrovolska O. Brilkov M. Madeleine N. Ødegård-Fougner Ø. Strømland Ø. Martin S. R. (2020). The Arabidopsis (ASHH2) CW Domain Binds Monomethylated K4 of the Histone H3 Tail through Conformational Selection. FEBS J. 287 (20), 4458–4480. 10.1111/febs.15256 32083791

[B11] Dobrovolska O. Bril’kov M. Ødegård-Fougner Ø. Aasland R. Halskau Ø. (2018). 1H, 13C, and 15N Resonance Assignments of CW Domain of the N-Methyltransferase ASHH2 Free and Bound to the Mono-, Di- and Tri-Methylated Histone H3 Tail Peptides. Biomol. NMR Assignments 12 (1), 215–220. 10.1007/s12104-018-9811-x 29453713

[B12] Dong G. Ma D.-P. Li J. (2008). The Histone Methyltransferase SDG8 Regulates Shoot Branching in Arabidopsis. Biochem. Biophysical Res. Commun. 373, 659–664. 10.1016/j.bbrc.2008.06.096 18602372

[B13] Du X. Li Y. Xia Y. L. Ai S. M. Liang J. Sang P. (2016). Insights into Protein-Ligand Interactions: Mechanisms, Models, and Methods. Int. J. Mol. Sci. 17 (2), 144. 10.3390/ijms17020144 PMC4783878 26821017

[B14] Duy C. Fitter J. (2006). How Aggregation and Conformational Scrambling of Unfolded States Govern Fluorescence Emission Spectra. Biophysical J. 90, 3704–3711. 10.1529/biophysj.105.078980 PMC1440751 16500981

[B15] Farber P. J. Mittermaier A. (2015). Relaxation Dispersion NMR Spectroscopy for the Study of Protein Allostery. Biophys. Rev. 7, 191–200. 10.1007/s12551-015-0166-6 28510170 PMC5425744

[B16] Ferrante A. Gorski J. (2012). Enthalpy-Entropy Compensation and Cooperativity as Thermodynamic Epiphenomena of Structural Flexibility in Ligand-Receptor Interactions. J. Mol. Biol. 417, 454–467. 10.1016/j.jmb.2012.01.057 22342886 PMC3349339

[B17] Gianni S. Jemth P. (2019). Affinity Versus Specificity in Coupled Binding and Folding Reactions. Protein Eng. Des. Sel 32, 355–357. 10.1093/protein/gzz020 31397874

[B18] Grini P. E. Thorstensen T. Alm V. Vizcay-Barrena G. Windju S. S. Jørstad T. S. (2009). The ASH1 HOMOLOG 2 (ASHH2) Histone H3 Methyltransferase Is Required for Ovule and Anther Development in Arabidopsis. PLoS One 4, e7817. 10.1371/journal.pone.0007817 19915673 PMC2772814

[B19] He F. Umehara T. Saito K. Harada T. Watanabe S. Yabuki T. (2010). Structural Insight into the Zinc finger CW Domain as a Histone Modification Reader. Structure 18, 1127–1139. 10.1016/j.str.2010.06.012 20826339

[B20] Homans S. W. (2007). “Dynamics and Thermodynamics of Ligand–Protein Interactions,” in Bioactive Conformation I. Editors Peters T. (Berlin, Heidelberg: Springer Berlin Heidelberg), 51–82.

[B21] Hoppmann V. Thorstensen T. Kristiansen P. E. Veiseth S. V. Rahman M. A. Finne K. (2011). The CW Domain, a New Histone Recognition Module in Chromatin Proteins. EMBO J. 30, 1939–1952. 10.1038/emboj.2011.108 21522130 PMC3098480

[B22] Iešmantavičius V. Dogan J. Jemth P. Teilum K. Kjaergaard M. (2014). Helical Propensity in an Intrinsically Disordered Protein Accelerates Ligand Binding. Angew. Chem. Int. Ed. Engl. 53, 1548–1551. 10.1002/anie.201307712 24449148

[B23] Keller R. L. J. (2005). Optimizing the Process of Nuclear Magnetic Resonance Spectrum Analysis and Computer Aided Resonance Assignmented. Zürich: ETH. Available at: https://www.research-collection.ethz.ch/bitstream/handle/20.500.11850/148938/eth-28223-01.pdf?sequence=1&isAllowed=y .

[B24] Kharchenko V. Nowakowski M. Jaremko M. Ejchart A. Jaremko Ł. (2020). Dynamic 15N{1H} NOE Measurements: A Tool for Studying Protein Dynamics. J. Biomol. NMR 74, 707–716. 10.1007/s10858-020-00346-6 32918646 PMC7701129

[B25] Ladbury J. E. (2010). Calorimetry as a Tool for Understanding Biomolecular Interactions and an Aid to Drug Design. Biochem. Soc. Trans. 38, 888–893. 10.1042/bst0380888 20658972

[B26] Lescop E. Schanda P. Brutscher B. (2007). A Set of BEST Triple-Resonance Experiments for Time-Optimized Protein Resonance Assignment. J. Magn. Reson. 187, 163–169. 10.1016/j.jmr.2007.04.002 17468025

[B27] Li H. Xie Y. Liu C. Liu S. (2014). Physicochemical Bases for Protein Folding, Dynamics, and Protein-Ligand Binding. Sci. China Life Sci. 57, 287–302. 10.1007/s11427-014-4617-2 24554472

[B28] Li S. Yen L. Pastor W. A. Johnston J. B. Du J. Shew C. J. (2016). Mouse MORC3 Is a GHKL ATPase that Localizes to H3K4me3 Marked Chromatin. Proc. Natl. Acad. Sci. USA 113, E5108–E5116. 10.1073/pnas.1609709113 27528681 PMC5024608

[B29] Liu Y. Huang Y. (2018). Uncovering the Mechanistic Basis for Specific Recognition of Monomethylated H3K4 by the CW Domain of Arabidopsis Histone Methyltransferase SDG8. J. Biol. Chem. 293 (17), 6470–6481. 10.1074/jbc.ra117.001390 29496997 PMC5925821

[B30] Liu Y. Tempel W. Zhang Q. Liang X. Loppnau P. Qin S. (2016). Family-Wide Characterization of Histone Binding Abilities of Human CW Domain-Containing Proteins. J. Biol. Chem. 291, 9000–9013. 10.1074/jbc.m116.718973 26933034 PMC4861470

[B31] Maciejewski M. W. Schuyler A. D. Gryk M. R. Moraru I. I. Romero P. R. Ulrich E. L. (2017). NMRbox: A Resource for Biomolecular NMR Computation. Biophysical J. 112, 1529–1534. 10.1016/j.bpj.2017.03.011 PMC5406371 28445744

[B32] Matulis D. Kranz J. K. Salemme F. R. Todd M. J. (2005). Thermodynamic Stability of Carbonic Anhydrase: Measurements of Binding Affinity and Stoichiometry Using ThermoFluor. Biochemistry 44, 5258–5266. 10.1021/bi048135v 15794662

[B33] Mellor J. (2006). It Takes a PHD to Read the Histone Code. Cell 126, 22–24. 10.1016/j.cell.2006.06.028 16839870

[B34] Nguyen H. Park J. Kang S. Kim M. (2015). Surface Plasmon Resonance: A Versatile Technique for Biosensor Applications. Sensors 15, 10481–10510. 10.3390/s150510481 25951336 PMC4481982

[B35] Patel D. J. (2016). A Structural Perspective on Readout of Epigenetic Histone and DNA Methylation Marks. Cold Spring Harb Perspect. Biol. 8, a018754. 10.1101/cshperspect.a018754 26931326 PMC4772102

[B36] Perozzo R. Folkers G. Scapozza L. (2004). Thermodynamics of Protein-Ligand Interactions: History, Presence, and Future Aspects. J. Receptors Signal Transduction 24, 1–52. 10.1081/rrs-120037896 15344878

[B37] Perry J. Zhao Y. (2003). The CW Domain, a Structural Module Shared Amongst Vertebrates, Vertebrate-Infecting Parasites and Higher Plants. Trends Biochem. Sci. 28, 576–580. 10.1016/j.tibs.2003.09.007 14607086

[B38] Pettersen E. F. Goddard T. D. Huang C. C. Couch G. S. Greenblatt D. M. Meng E. C. (2004). UCSF Chimera-Aa Visualization System for Exploratory Research and Analysis. J. Comput. Chem. 25, 1605–1612. 10.1002/jcc.20084 15264254

[B39] Piserchio A. Pellegrini M. Mehta S. Blackman S. M. Garcia E. P. Marshall J. (2002). The PDZ1 Domain of SAP90. Characterization of Structure and Binding. J. Biol. Chem. 277, 6967–6973. 10.1074/jbc.m109453200 11744724

[B40] Questa J. I. Rius S. P. Casadevall R. Casati P. (2016). ZmMBD101 Is a DNA-Binding Protein that maintainsMutatorelements Chromatin in a Repressive State in maize. Plant Cel Environ. 39, 174–184. 10.1111/pce.12604 26147461

[B41] Sanchez R. Zhou M. M. (2011). The PHD Finger: A Versatile Epigenome Reader. Trends Biochem. Sci. 36, 364–372. 10.1016/j.tibs.2011.03.005 21514168 PMC3130114

[B42] Schleucher J. Schwendinger M. Sattler M. Schmidt P. Schedletzky O. Glaser S. J. (1994). A General Enhancement Scheme in Heteronuclear Multidimensional NMR Employing Pulsed Field Gradients. J. Biomol. NMR 4, 301–306. 10.1007/BF00175254 8019138

[B43] Schneider R. Maurin D. Communie G. Kragelj J. Hansen D. F. Ruigrok R. W. H. (2015). Visualizing the Molecular Recognition Trajectory of an Intrinsically Disordered Protein Using Multinuclear Relaxation Dispersion NMR. J. Am. Chem. Soc. 137, 1220–1229. 10.1021/ja511066q 25551399

[B44] Schuck P. Zhao H. (2010). The Role of Mass Transport Limitation and Surface Heterogeneity in the Biophysical Characterization of Macromolecular Binding Processes by SPR Biosensing. Methods Mol. Biol. 627, 15–54. 10.1007/978-1-60761-670-2_2 20217612 PMC4134667

[B45] Shi Y. Lan F. Matson C. Mulligan P. Whetstine J. R. Cole P. A. (2004). Histone Demethylation Mediated by the Nuclear Amine Oxidase Homolog LSD1. Cell 119, 941–953. 10.1016/j.cell.2004.12.012 15620353

[B46] Steiner S. Magno A. Huang D. Caflisch A. (2013). Does Bromodomain Flexibility Influence Histone Recognition? FEBS Lett. 587, 2158–2163. 10.1016/j.febslet.2013.05.032 23711371

[B47] Taverna S. D. Li H. Ruthenburg A. J. Allis C. D. Patel D. J. (2007). How Chromatin-Binding Modules Interpret Histone Modifications: Lessons from Professional Pocket Pickers. Nat. Struct. Mol. Biol. 14, 1025–1040. 10.1038/nsmb1338 17984965 PMC4691843

[B48] Tencer A. H. Cox K. L. Wright G. M. Zhang Y. Petell C. J. Klein B. J. (2020). Molecular Mechanism of the MORC4 ATPase Activation. Nat. Commun. 11, 5466. 10.1038/s41467-020-19278-8 33122719 PMC7596504

[B49] Teske K. A. Hadden M. K. (2017). Methyllysine Binding Domains: Structural Insight and Small Molecule Probe Development. Eur. J. Med. Chem. 136, 14–35. 10.1016/j.ejmech.2017.04.047 28478342

[B50] Tochio H. Hung F. Li M. Bredt D. S. Zhang M. (2000). Solution Structure and Backbone Dynamics of the Second PDZ Domain of Postsynaptic Density-95. J. Mol. Biol. 295, 225–237. 10.1006/jmbi.1999.3350 10623522

[B51] Tsai C.-J. Kumar S. Ma B. Nussinov R. (1999). Folding Funnels, Binding Funnels, and Protein Function. Protein Sci. 8, 1181–1190. 10.1110/ps.8.6.1181 10386868 PMC2144348

[B52] Wang Y. Reddy B. Thompson J. Wang H. Noma K.-i. Yates J. R. (2009). Regulation of Set9-Mediated H4K20 Methylation by a PWWP Domain Protein. Mol. Cel 33, 428–437. 10.1016/j.molcel.2009.02.002 PMC2673476 19250904

[B53] Waterhouse A. M. Procter J. B. Martin D. M. A. Clamp M. Barton G. J. (2009). Jalview Version 2--a Multiple Sequence Alignment Editor and Analysis Workbench. Bioinformatics 25, 1189–1191. 10.1093/bioinformatics/btp033 19151095 PMC2672624

[B54] Xu L. Zhao Z. Dong A. Soubigou-Taconnat L. Renou J.-P. Steinmetz A. (2008). Di- and Tri- but Not Monomethylation on Histone H3 Lysine 36 marks Active Transcription of Genes Involved in Flowering Time Regulation and Other Processes in *Arabidopsis T* . Mol. Cel Biol 28, 1348–1360. 10.1128/mcb.01607-07 PMC2258740 18070919

[B55] Yang Z. Jiang J. Stewart D. M. Qi S. Yamane K. Li J. (2010). AOF1 Is a Histone H3K4 Demethylase Possessing Demethylase Activity-independent Repression Function. Cell Res 20, 276–287. 10.1038/cr.2010.12 20101264 PMC4106039

[B56] Ye Y. Godzik A. (2004). FATCAT: A Web Server for Flexible Structure Comparison and Structure Similarity Searching. Nucleic Acids Res. 32, W582–W585. 10.1093/nar/gkh430 15215455 PMC441568

[B57] Zhang Q. Qi S. Xu M. Yu L. Tao Y. Deng Z. (2013). Structure-Function Analysis Reveals a Novel Mechanism for Regulation of Histone Demethylase LSD2/AOF1/KDM1b. Cel Res 23, 225–241. 10.1038/cr.2012.177 PMC3567814 23266887

[B58] Zhang Y. Klein B. J. Cox K. L. Bertulat B. Tencer A. H. Holden M. R. (2019). Mechanism for Autoinhibition and Activation of the MORC3 ATPase. Proc. Natl. Acad. Sci. U S A. 116 (13), 6111–6119. 10.1073/pnas.1819524116 30850548 PMC6442546

[B59] Zhu J. Qi R. Liu Y. Zhao L. Han W. (2019). Mechanistic Insights into the Effect of Ligands on Structural Stability and Selectivity of Sulfotransferase 2A1 (SULT2A1). ACS Omega 4, 22021–22034. 10.1021/acsomega.9b03136 31891082 PMC6933797

