# Supplementary material for: Binding Specificity of ASHH2 CW Domain Toward H3K4me1 Ligand Is Coupled to Its Structural Stability Through Its α1-Helix
Source: Front Mol Biosci. 2022 Apr 13;9:763750. doi: 10.3389/fmolb.2022.763750 (PMC9043364; doi:10.3389/fmolb.2022.763750)
Supplement: Supplementary file 1 [file DataSheet1.docx]

## SUPPLEMENTARY

### Table S1. List of primers.

| Primer^a^ | Sequence^b^ |
| --- | --- |
| ASHH2-CW-D870A FW  ASHH2-CW-D870A RV  ASHH2-CW-insC866-SFPN-C871 FW  ASHH2-CW-insC866-SFPN-C871 RV  ASHH2-CW-I915A FW  ASHH2-CW I915A RV  ASHH2-CW L919A FW  ASHH2-CW L919A RV | 5’-TCGATGTGATgccTGCTTTAAATGGCGAC  5’-ACCCAGGCACTCTCTGTG  5’-cctaacTGCTTTAAATGGCGACGAATAC  5’-aaatgaACATCGAACCCAGGCACT  5’-AGAGTTGGGCATAGGACAGGATG  5’-TCATTagcTTCTTCATTTGACATCTCTTGAGATTTTGAGC  5’-AGAGgctGGCATAGGACAGGATGAAG  5’-TCATTAATTTCTTCATTTGACATCTCTTGAG |

^a^FW and RV indicate forward and reverse primers, respectively.

^b^Lower case indicates mutated codons.

### Table S2. Histone mimicking peptides.

| Name | Sequence | Manufacturer |
| --- | --- | --- |
| H3  H3K4me1  H3K4me2  H3K4me3 | ARTKQTARY*  ARTK(me1)QTARY*  ARTK(me2)QTARY*  ARTK(me3)QTARY* | LifeTein LLC (95% purity)  LifeTein LLC (95% purity)  LifeTein LLC (95% purity)  LifeTein LLC (95% purity) |

*An additional tyrosine was introduced for concentration determination by absorption at 280 nm (NanoDrop), and an extinction coefficient of 1490 M^-1^ cm^-1^ was used.

### Table S3. Summary of ITC data.

| Ligand | Temperature, K | ΔH, kJ/mol | TΔS, kJ/mol | ΔG, kJ/mol | K_d_, μM |
| --- | --- | --- | --- | --- | --- |
| CW wild type  H3K4me1  H3K4me2  H3K4me3  D870A  H3K4me1  H3K4me2  H3K4me3  L919A  H3K4me1 | 298.15  298.15  298.15  298.15  298.15  298.15  298.15 | -89.25±8.58  -83.64±3.11  -60.14±1.38  -79.22±4.81  -93.33±3.65  -57.47±2.37  -76.92±2.31 | -57.37±7.14  -53.18±3.23  -32.47±1.49  -45.70±4.10  -64.03±3.59  -30.45±2.65  -48.84±1.97 | -31.88±2.78  -30.46±0.15  -27.67±0.13  -33.52±0.71  -29.29±0.23  -27.02±0.29  -28.08±0.42 | 1.31±0.32  4.61±0.28  14.18±0.74  1.37±0.32  7.48±0.72  18.53±2.17  12.17±2.10 |


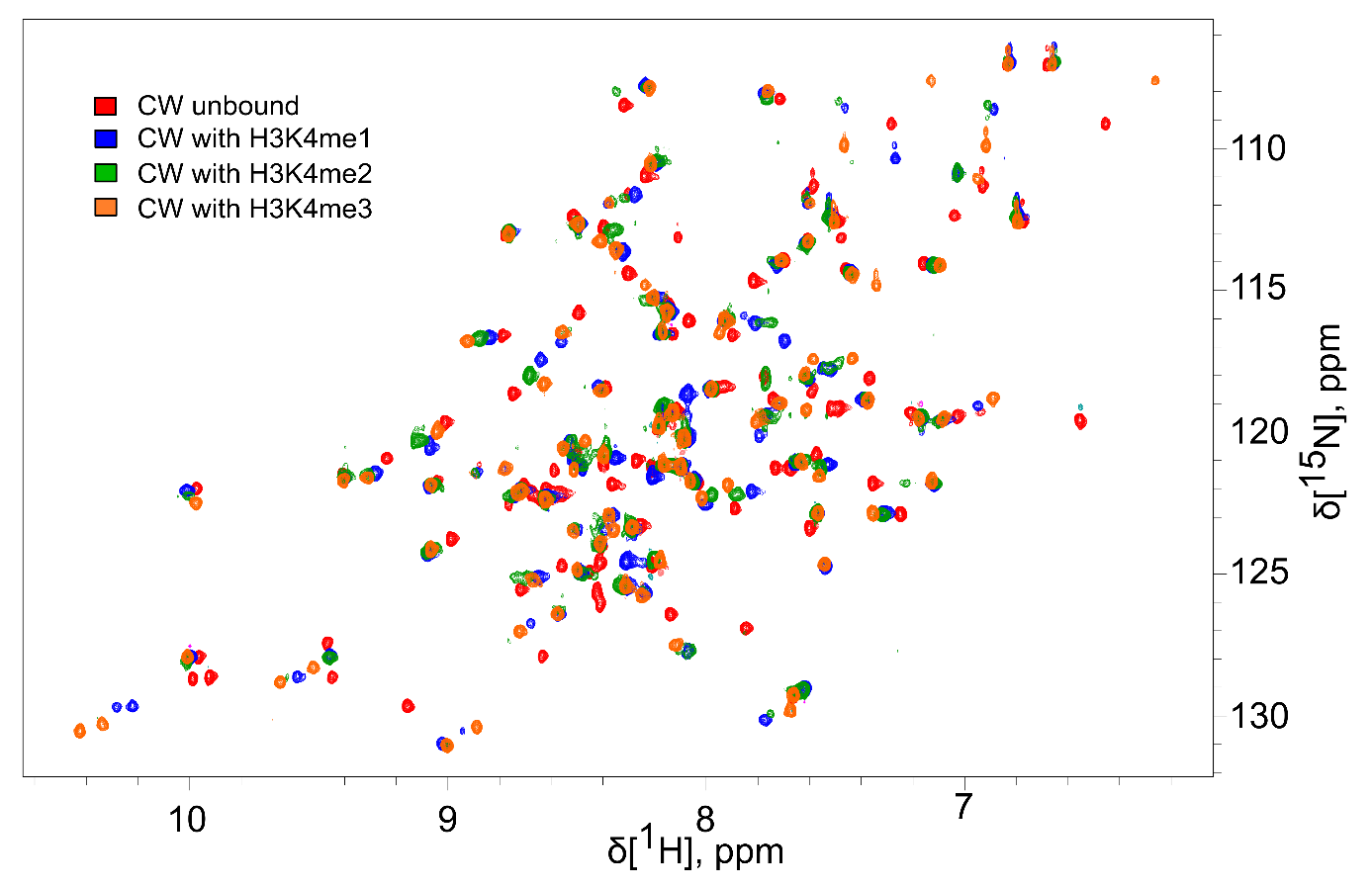


Figure S1.

Overlaid HSQC spectra of CW wild type in free state (red) and bound to H3K4me1 (blue), H3K4me2 (green) and H3K4me3 (orange) peptides.


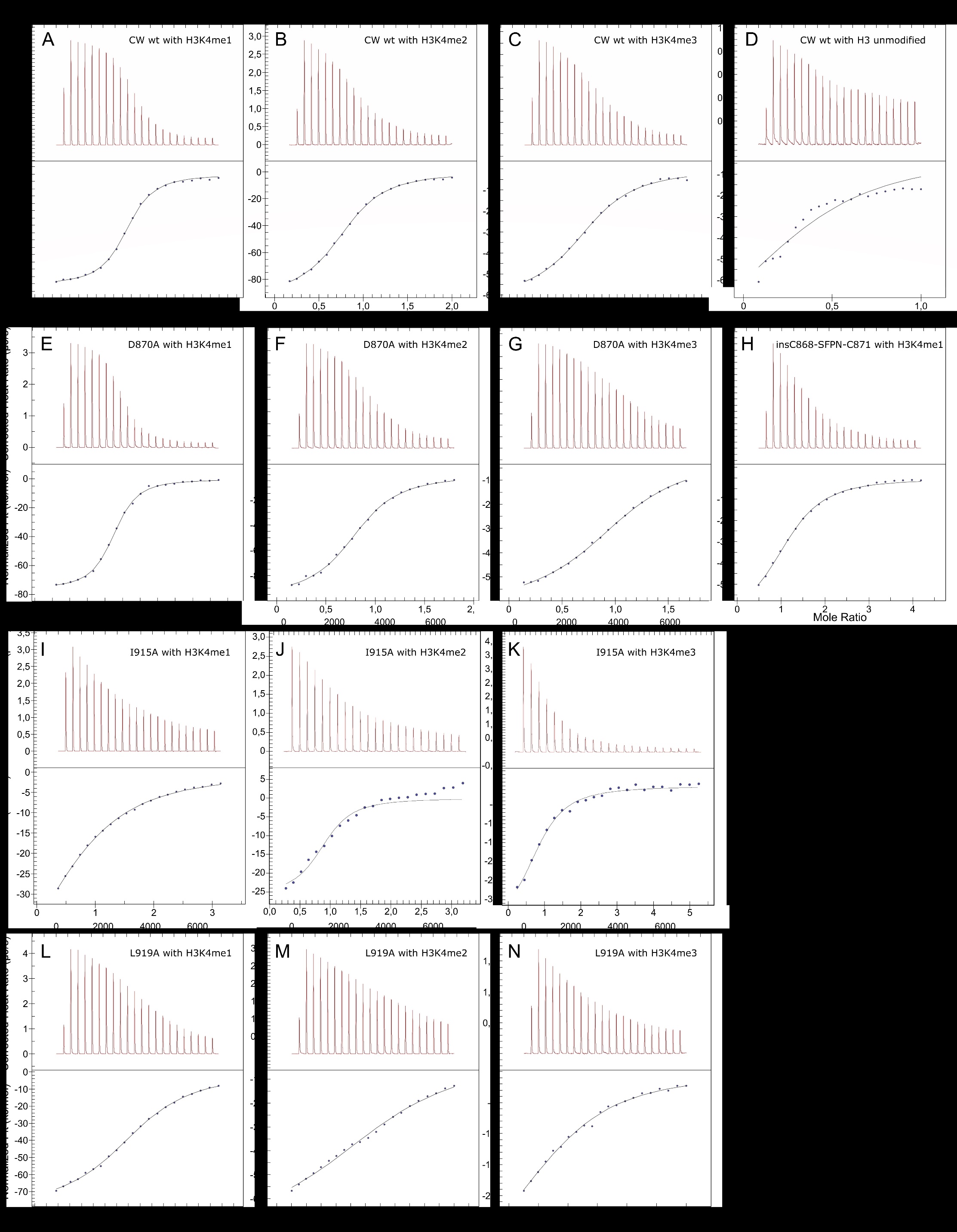


Figure S2

Titration curves and integrated data of: A-C – CW wt with H3K4me1/2/3 peptides; D-F – D870A with H3K4me1/2/3 peptides; G-I – L919A with H3K4me1/2/3 peptides; J – CW wt with H3 unmodified peptide; K – insC866-SFPN-C871 with H3K4me1 peptide; L – I915A with H3K4me1 peptide. In H-L no sigmoidal curve could be fitted.
